# Thaumarchaea Genome Sequences from a High Arctic Active Layer

**DOI:** 10.1128/MRA.00326-20

**Published:** 2020-05-21

**Authors:** Emily Wei-Hsin Sun, Sassan Hajirezaie, Mackenzie Dooner, Tatiana A. Vishnivetskaya, Alice Layton, Archana Chauhan, Susan M. Pfiffner, Lyle G. Whyte, Tullis C. Onstott, Maggie C. Y. Lau

**Affiliations:** a Department of Civil and Environmental Engineering, Princeton University, Princeton, New Jersey, USA; b Department of Humanistic Studies, Princeton University, Princeton, New Jersey, USA; c Center for Environmental Biotechnology, University of Tennessee, Knoxville, Tennessee, USA; d Department of Natural Resource Sciences, McGill University, Québec, Canada; e Department of Geosciences, Princeton University, Princeton, New Jersey, USA; f Laboratory of Extraterrestrial Ocean Systems, Institute of Deep-sea Science and Engineering, Chinese Academy of Sciences, Sanya, China; Queens College

## Abstract

The role of archaeal ammonia oxidizers often exceeds that of bacterial ammonia oxidizers in marine and terrestrial environments but has been understudied in permafrost, where thawing has the potential to release ammonia. Here, three thaumarchaea genomes were assembled and annotated from metagenomic data sets from carbon-poor Canadian High Arctic active-layer cryosols.

## ANNOUNCEMENT

Recent studies have shown that ammonia-oxidizing archaea (AOA) often outnumber ammonia-oxidizing bacteria (AOB) (1–3) and that AOA do the lion’s share of ammonia oxidation in terrestrial soils (3–9). However, their potential role in both fixed nitrogen losses and ozone-depleting nitric oxide or nitrous oxide release and their taxonomic diversity are poorly understood in terrestrial permafrost ecosystems (10–12). Here, we report three draft genome sequences of thaumarchaea that are potential chemolithoautotrophic ammonia oxidizers and may play a potentially significant role in the nitrogen cycle of the Canadian High Arctic.

These three genomes were constructed through the analysis of 21 metagenomes from mineral cryosols at 5-cm depth retrieved in our previously published studies (13, 14). Metagenomic libraries were prepared using the Illumina Nextera DNA library preparation kit (Illumina, Inc., San Diego, CA), followed by 100-bp paired-end DNA sequencing on an Illumina HiSeq 2000 platform (13). A total of 498,483,227 forward and reverse reads were filtered separately for quality using tools available on the Princeton University Galaxy server as follows: reads with 90% of the bases with a Phred score of <30 were removed using “filter by quality” v1.0.0; Nextera transposase adaptor sequences were trimmed using cutadapt v1.6; FASTQ Trimmer v1.0.0 was used to remove the last five bases at the 3′ end; and trimmed reads with fewer than 50 nucleotides (nt) were removed. One of the paired reads may be discarded, and the remaining read is referred to as a single read. Default parameters were used for all software unless otherwise specified.

Individual metagenomes were assembled using IDBA-UD v1.1.1 (15), and the scaffolds were sorted into taxonomic bins using MetaBAT v0.32.4 in “very specific” mode (16). The bins recovered from each of the 21 metagenomes were analyzed using CheckM v1.0.7 to assess the quality and taxonomy (17). The taxa appearing multiple times across the 21 metagenomes included 10 *Nitrosopumilales* bins within the phylum *Thaumarchaeota* (completeness, 3.88 to 97.73%; contamination, 0 to 2.02%). Of these, three *Nitrosopumilales* bins were 71.62 to 97.73% complete, so the quality reads from the three corresponding metagenomes (NCBI Sequence Read Archive accession numbers SRR1586318, SRR1586310, and SRR1586268), which were mapped onto their respective genome bins using Bowtie 2 v2.3.2 (18), were extracted and reassembled separately using IDBA-UD v1.1.1. The number of mapped reads was used to calculate the depth of the coverage. The quality and taxonomy of the draft genomes were evaluated using CheckM v1.0.7. The statistics of these three *Nitrosopumilales* draft genome sequences are presented in Table 1.

**TABLE 1:** Genome statistics for AHI_AL_Thaum01 to AHI_AL_Thaum03

Characteristic	Data for strain:		
	AHI_AL_Thaum01	AHI_AL_Thaum02	AHI_AL_Thaum03
Metagenome data set from which the draft genome originated (SRA accession no.)	SRR1586268	SRR1586310	SRR1586318
No. of paired-end reads in the original data set	150,218,712	34,785,144	38,551,334
Quality-filtered reads available for the initial assembly			
No. of paired reads (average read length [nt])	24,966,724^*a*^ (96)	13,300,133 (93)	18,935,562 (88)
No. of single reads (average read length [nt])	31,932,629^*a*^ (96)	9,420,119 (93)	9,420,119 (89)
Quality-filtered reads extracted for reassembly			
No. of paired reads (average read length [nt])	132,263 (96)	195,388 (93)	184,721 (88)
No. of single reads (average read length [nt])	76,789 (96)	36,635 (92)	22,553 (86)
Genome size (bp)	1,442,500	2,538,969	1,925,816
No. of contigs	578	521	602
GC content (%)	39.6	39.3	39.3
Minimum contig length (bp)	1,000	1,000	1,003
*N*_50_ (bp)	2,965	6,780	3,871
Completeness (%)	62.73	98.71	84.47
Contamination (%)	2.18	2.43	2.99
No. of predicted coding genes^*b*^	1,495	2,587	2,014
Mean base coverage (×)	12.07	8.48	9.49
GenBank accession no.	WJXC00000000	WJXE00000000	WJXD00000000

a Sample SRR1586268 was assembled using only the paired reads because the inclusion of single reads failed to run due to an IDBA-UD segmentation fault that was not resolved.

b Annotated using the NCBI Prokaryotic Genome Annotation Pipeline v4.10.

Prodigal (Prokaryotic Dynamic Programming Genefinding Algorithm) v2.6.3 (19) was employed for gene prediction. Genes were annotated using the NCBI Prokaryotic Genome Annotation Pipeline v4.10 (PGAP) (20), and the metabolic pathways were analyzed using the KEGG Automatic Annotation Server (KAAS) v2.1 (21). The results revealed genes belonging to the 3-hydroxypropanoate/4-hydroxybutanoate carbon fixation pathway, ammonia monooxygenase subunits A, B, and C (*amoCAB* gene), and nitrite reductase (*nirK* gene). Like in the ammonia-oxidizer Nitrosopumilus maritimus SCM1 (22), the gene encoding hydroxylamine oxidoreductase (HAO), the key enzyme in the conversion of hydroxylamine to nitrite, is absent in all three genomes. The enzyme 4-hydroxybutyryl-coenzyme A dehydratase (4-HCD) identified in the three thaumarchaea genomes has been proposed as an environmental marker for autotrophic thaumarchaea (23).

### Data availability.

This genome assembly project and the three high-completion genome sequences have been deposited at NCBI GenBank under the accession numbers WJXC00000000, WJXD00000000, and WJXE00000000 (BioSample numbers SAMN11973980, SAMN11973981, and SAMN11973982 and BioProject number PRJNA548371). The versions described in this paper are the first versions, WJXC01000000, WJXD01000000, and WJXE01000000. The raw reads were deposited at the NCBI Sequence Read Archive under the accession number SRP047512 (13).
